# Existential anxiety about artificial intelligence (AI)- is it the end of humanity era or a new chapter in the human revolution: questionnaire-based observational study

**DOI:** 10.3389/fpsyt.2024.1368122

**Published:** 2024-04-09

**Authors:** Joud Mohammed Alkhalifah, Abdulrahman Mohammed Bedaiwi, Narmeen Shaikh, Waleed Seddiq, Sultan Ayoub Meo

**Affiliations:** ^1^ College of Medicine, King Saud University, Riyadh, Saudi Arabia; ^2^ Division of Cardiovascular Medicine, Radcliffe Department of Medicine, University of Oxford, Oxford, United Kingdom; ^3^ Department of Physiology, College of Medicine, King Saud University, Riyadh, Saudi Arabia

**Keywords:** artificial intelligence, anxiety, fear, existential anxiety, human revolution

## Abstract

**Background:**

Existential anxiety can profoundly affect an individual, influencing their perceptions, behaviours, sense of well-being, academic performance, and decisions. Integrating artificial intelligence into society has elicited complex public reactions, marked by appreciation and concern, with its acceptance varying across demographics and influenced by factors such as age, gender, and prior AI experiences. This study aimed to investigate the existential anxiety about artificial intelligence (AI) in public in Saudi Arabia.

**Methods:**

The present questionnaire-based observational, analytical cross-sectional study with a structured, self-administered survey was conducted via Google Forms, using a scale to assess the existential anxiety levels induced by the recent development of AI. The study encompassed a diverse population with a sample size of 300 participants.

**Results:**

This study’s findings revealed a high prevalence of existential anxieties related to the rapid advancements in AI. Key concerns included the fear of death (96% of participants), fate’s unpredictability (86.3%), a sense of emptiness (79%), anxiety about meaninglessness (92.7%), guilt over potential AI-related catastrophes (87.7%), and fear of condemnation due to ethical dilemmas in AI (93%), highlighting widespread apprehensions about humanity’s future in an AI-dominated era.

**Conclusion:**

The public has concerns including unpredictability, a sense of emptiness, anxiety, guilt over potential AI-related catastrophes, and fear of condemnation due to ethical dilemmas in AI, highlighting widespread apprehensions about humanity’s future in an AI-dominated era. The results indicate that there is a need for a multidisciplinary strategy to address the existential anxieties in the AI era. The strategic approach must blend technological advancements with psychological, philosophical, and ethical insights, underscoring the significance of human values in an increasingly technology-driven world.

## Introduction

Existential anxiety is the term used to describe the intense disquiet or discomfort experienced when faced with the inherent uncertainties of existence and the certainty of death. Major world crises, like the COVID-19 pandemic, have been linked to increased existential fears, as people grapple with the fragility of life and their mortality (1). This type of anxiety can significantly impact human behaviour, influencing academic performance (2), political dynamics during crises (3), and health-related problems (4).

The most recent developments in artificial intelligence (AI) have also impacted several academic and industrial sectors, notably education, medicine, and transportation. Public attitudes towards AI are complex, characterized by a mix of appreciation for its potential benefits and concerns about its implications (5). The transformative power of AI is recognized, but concerns about privacy, trust, and the consequences of AI decisions in high-stakes situations are prevalent (6, 7). Moreover, understanding and acceptance of AI can vary significantly based on demographic factors such as gender, age, and prior experience with AI (6).

Artificial intelligence has been integrated into many parts of society, revolutionizing industries, and significantly influencing public life. As AI technology becomes more commonplace, public concerns regarding its effects are growing. Studies have shown that while AI may enhance urban decision-making and public services, it can also induce technological anxiety, particularly when perceived internal threats to AI and the Internet of Things (IoT) are present (5). Diverse opinions about AI, influenced by factors like gender, age, and past AI experiences, have been observed (6). Therefore, understanding these concerns and addressing them is crucial for the responsible deployment of AI (7). While we tried to incorporate the literature to better understand the findings, we noticed a huge deficiency in this area, up to our knowledge we could not find a similar study scope that was done elsewhere in the entire medical literature. On the contrary many articles were written by concerned honest journalists around the world but the medical literature is falling behind on the matter; thus, study aimed to investigate the existential anxiety about artificial intelligence (AI) in public in Saudi Arabia.

## Subjects and methods

### Study design and settings

The present questionnaire-based observational–analytical cross-sectional study was conducted in the Department of Physiology, College of Medicine, King Saud University, Riyadh, Saudi Arabia, from July–December 2023.

### Inclusion and exclusion criteria and data collection

The targeted population of the current study was the common population above the age of 18 years old. Participants with a prior diagnosis of any mental health disorder were systematically excluded from the study, to eliminate the confounder effect of anxiety due to underlying medical conditions. This exclusion criterion was implemented at the outset of the questionnaire; respondents affirming a history of mental health disease in response to the initial question were immediately disqualified, resulting in the termination of their participation in the survey. A well-structured, self-administered, validated electronic questionnaire survey in the English Language was conducted via Google Forms on social media, using a scale to assess their existential anxiety levels induced by the recent development of AI. The demographic data of the study population were obtained through an online survey via the same Google forms. The selection of samples was made using the convenient sampling technique. For data collection, the investigators were assigned to ensure that the data was inclusive. The power formula was employed to calculate the sample size; as per an earlier published study (8).

### Existential anxiety scale

Our study focused on identifying prevalent existential anxiety (EA) concerns among participants, as per Tillich’s theory of EA (9). These included worries about death, fate, meaninglessness, emptiness, condemnation, and guilt (9).​ The EA questionnaire (EAQ), as developed by Weems et al., serves as a valuable tool for the assessment of Experiential Avoidance (EA) within study participants (10). Comprising 13 items, this scale employs a binary response format, requiring respondents to answer either “yes” or “no” based on their agreement with the posed questions. Notably, each concern addressed in the questionnaire is accompanied by two questions: one that is positively scored, denoting the presence of EA when answered affirmatively, and another that is negatively scored, signifying the absence of EA with an affirmative response. Regarding the concept of fate, the questionnaire includes three items: one positively scored and two negatively scored. Weems et al. (9) evaluated the EAQ’s psychometric properties and reported satisfactory results in terms of internal consistency, with a coefficient alpha (α) of 0.71, as well as test-retest reliability, with a correlation coefficient (r) of 0.72 at a two-week interval, with statistical significance (P < 0.001) (8). The total EA score for an individual is computed by summing the number of items endorsing the presence of EA, yielding a score ranging from 0 to 13. It is worth noting that the EAQ has garnered substantial attention within the research community for its robustness and versatility in measuring EA, demonstrating its validity across diverse populations (10, 11). Consequently, considering the various questionnaires developed for EA assessment, the EAQ has emerged as the instrument of choice for our specific study population, underlining its suitability and reliability as an indispensable tool in EA research. We employed the instrument exactly with no changes but adding the prefix (In terms of recent Artificial intelligence development) preceding each statement of the scale.

### Statistical analysis

The study findings were analyzed using the SPSS software version 26.0 for Mac. The demographic variables, including “age, gender, and occupation status were reported using frequency and percentage. The total response score was summarized and reported using mean and standard deviation (Mean SD). The comparisons between the variables with demographic and clinical factors were analyzed using the Chi-square test with a degree of freedom (df). A *p*-value <0.05 was considered as significant.”

## Results

### Demographics

Our study encompassed a diverse population with a sample size of n=300, Among them, 215 individuals (71.7%) were Saudi nationals, while 85 participants (28.3%) were of non-Saudi nationality. Gender distribution was balanced, with 122 males (40.7%) and 178 females (59.3%). Age-wise, the study showed a significant representation of younger individuals, with those aged 20-30 forming the largest group at 151 participants (50.3%). The 31-40 age group accounted for 73 individuals (24.3%), followed by 41-50 years at 23 participants (7.7%), 51-60 years at 23 participants (7.7%), and those aged 61 and above represented by a smaller fraction of 6 individuals (2%). Marital status varied, with 211 participants (70.3%) being single, 7 (2.3%) engaged, 76 (25.3%) married, and 6 (2%) either divorced or widowed. In terms of employment status, a majority were employed or on payroll (193 participants or 64.3%), followed by students or unemployed individuals (93 participants or 31%), and a smaller segment of retirees (14 participants or 4.7%). The work sector representation was in government (168 participants or 56%), with private sector employees constituting 39 individuals (13%) and students or unemployed participants making up 93 individuals (31%). When considering years of experience in work or study, those with 1-5 years formed the largest group at 97 individuals (32.3%), followed by those with 6-10 years of experience at 32 participants (10.7%), 11-15 years at 23 participants (7.7%), 16-20 years at 7 participants (2.3%), and those with 21 years or more at 45 participants (15%). The occupational distribution among the participants was diverse, with a significant presence in the health, science, and engineering sectors, comprising 156 individuals or 52% of the total. The education sector, including faculty, researchers, and government employees, was also well-represented with 73 participants, accounting for 24.3%. Additionally, the humanities, arts, literature, culture, entertainment, and communication fields had 29 participants, making up 9.7% of the total. The IT and technology sector had a notable presence with 18 participants, contributing to 6% of the demographic. Business and law combined had 14 participants, representing 4.7%, while freelancers accounted for 10 participants or 3.3% of the total (**Table 1**). This demographic composition offers valuable insights into the diverse backgrounds of individuals engaged in discussions about the impact of AI on the future of humanity.

**Table 1:** Socio-demographics characteristics of the respondents.

Characteristic	n (%)
Nationality	
Saudi	215 (71.7)
Non-Saudi	85 (28.3)
Gender	
Male	122 (40.7)
Female	178 (59.3)
Age	
Younger than 20	73 (24.3)
20-30	151 (50.3)
31-40	23 (7.7)
41-50	23 (7.7)
51-60	24 (8)
61 and above	6 (2)
Marital Status	
Single	211 (70.3)
Engaged	7 (2.3)
Married	76 (25.3)
Divorced or widowed	6 (2)
Employment Status	
Employed/On payroll	97 (32.3)
Unemployed/Not on payroll	175 (58.3)
Retired	28 (9.3)
Work Sector	
Government	93 (31)
Private	39 (13)
Student/Unemployed	168 (56)
Years of Experience at Work/Study	
1-5 years	193 (64.3)
6-10 years	32 (10.7)
11-15 years	23 (7.7)
16-20 years	7 (2.3)
21 years or more	45 (15)
Occupation	
Health, science, and engineering	156 (52)
Education, Faculty, Researcher, Government	73 (24.3)
Humanities, Art, Literature, culture, entertainment	29 (9.7)
IT and technology	18 (6)
Business and law	14 (4.7)
Freelancers	10 (3.3)
**Total**	**300 (100)**

### Use of artificial intelligence

**Figure 1:**
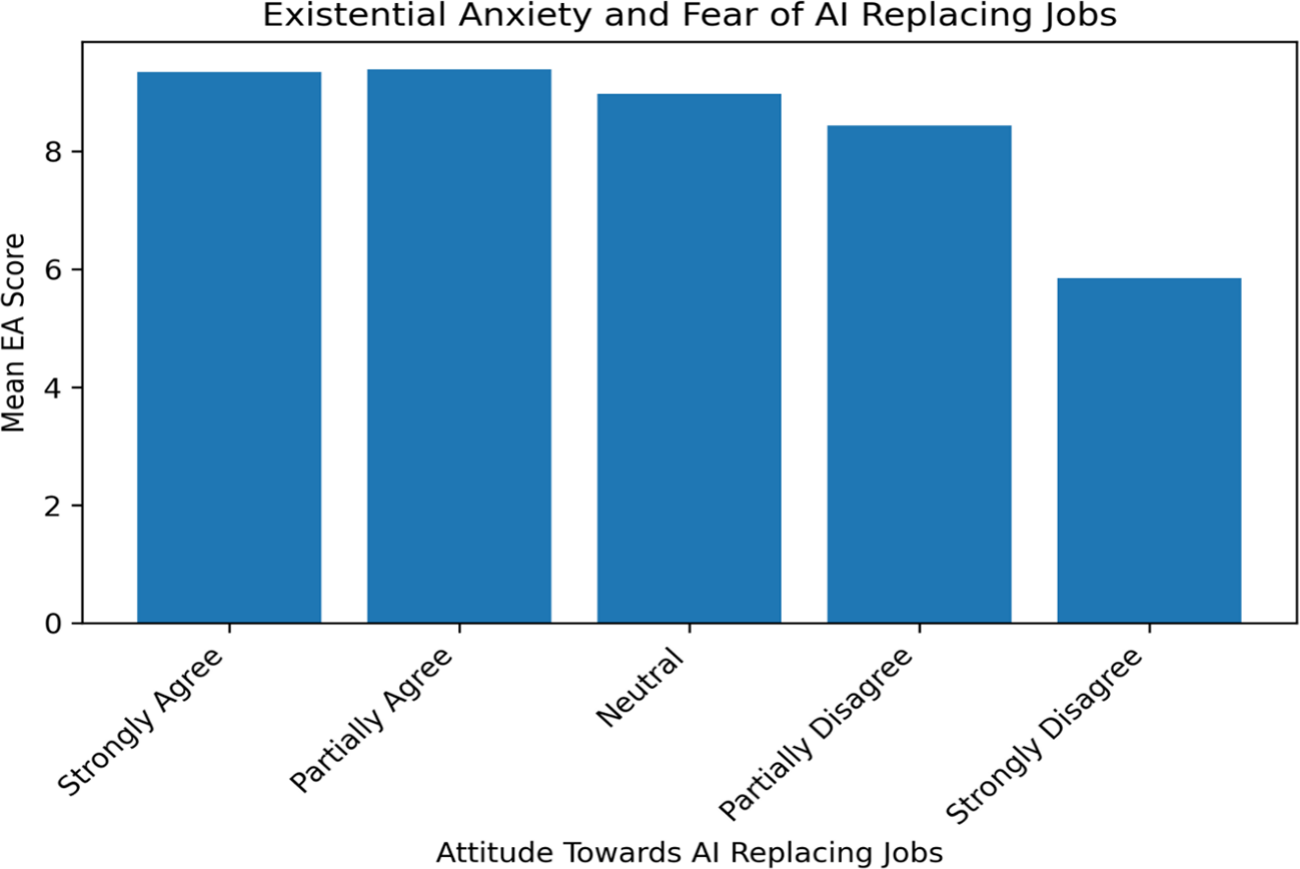
Existential anxiety and fear of AI replacing jobs.

The study participants exhibited a range of engagements and concerns as shown in **Table 2**. Regarding their awareness of AI technologies like Chat-GPT, 65 (21.7%) were both aware of and actively using these technologies, whereas 92 (30.7%) knew about them but had not engaged with them. The majority group of 147 (47.7%) remained completely unaware of such advancements. As for the frequency of AI usage in their careers, the responses varied, with 65 participants (21.7%) using AI rarely (1-3 times/month), 92 (30.7%) often (once weekly), 143 (47.7%) constantly (every other day), and 123 (41%) always (daily), highlighting the diverse integration of AI in different professions. When asked about the fear of AI replacing human jobs, 65 participants (21.7%) strongly agreed, 92 (30.7%) partially agreed, 143 (47.7%) were neutral, 123 (41%) partially disagreed, and 55 (18.3%) strongly disagreed. These numbers indicate a spectrum of existential anxiety, with varying levels of concern about AI’s impact on job security, reflecting a complex picture of how AI is perceived to influence the future of work and personal life (**Figure 1**).

**Table 2:** Questions regarding the use of AI.

Questions	*n (%)*
Are you aware of the recent development of Artificial Intelligence (AI), for example: Chat-GPT?	
Yes, and I have tried the chatbot	65 (21.7)
Yes, but I have not tried it yet	92 (30.7)
No	143 (47.7)
If you answered yes to the previous question, how often do you use AI in your career?	
Rarely (1-3 times/month)	50 (71.4)
Often (once weekly)	16 (18.3)
Constantly (every other day)	5 (7.3)
Always (daily)	2 (4.3)
I have a genuine fear that in the near future Artificial Intelligence may replace me at my job.	
Strongly Agree	65 (21.7)
Partially Agree	70 (23.3)
Neutral	65 (21.7)
Partially Disagree	71 (23.7)
Strongly Disagree	26 (8.7)

### Existential anxiety prevalence

We found a high prevalence of various existential concerns as demonstrated in **Tables 3**, **4**. The most common concern was the fear of death, experienced by 288 participants (96%), highlighting near-universal anxiety about mortality in the context of rapid AI advancements. Concerns about fate were also prominent, with 259 individuals (86.3%) expressing unease about the unpredictability and uncontrollability of life in an AI-driven future. The feeling of emptiness was reported by 237 participants (79%), indicating a sizable number of individuals grappling with a sense of void or lack of fulfilment. An overwhelming 278 (92.7%) experienced anxiety related to meaninglessness, reflecting apprehension about finding purpose in a rapidly evolving world. Guilt, stemming from the perceived human role in creating potentially apocalyptic AI, was reported by 263 participants (87.7%). Finally, fear of condemnation, potentially linked to ethical and moral dilemmas associated with AI, was felt by 279 individuals (93%). These findings underscore an elevated level of existential anxiety across various dimensions, pointing to widespread apprehensions about the future of humanity in the era of advanced AI technology.

**Table 3:** Existential anxiety questionnaire.

Item	Response	
	No	Yes
1. I often think about death, and this causes me anxiety. (D)	29 (9.7)	271 (90.3)
2. I am not anxious about fate because I am resigned to it. R (F)	250 (83.3)	50 (16.7)
3. I often feel anxious because I am worried that life may have no meaning. (M)	60 (20)	240 (80)
4. I am not worried about nor think about being guilty. R (G)	209 (69.7)	91 (30.3)
5. I often feel anxious because of feelings of guilt. (G)	65 (21.7)	235 (78.3)
6. I often feel anxious because I feel condemned. (C)	38 (12.7)	262 (87.3)
7. I never think about emptiness. R (E)	171 (57)	129 (43)
8. I often think that things that were once important in life are empty. (E)	143 (47.7)	157 (52.3)
9. I never feel anxious about being condemned. R (C)	230 (76.7)	70 (23.3)
10. I am not anxious about death because I am prepared for whatever it may bring. R(D)	185 (61.7)	115 (38.3)
11. I often think about fate, and it causes me to feel anxious. (F)	112 (37.3)	188 (62.7)
12. I am not anxious about fate because I am sure that things will work out. R (F)	236 (78.7)	64 (21.3)
13. I know life has meaning. R (M)	247 (82.3)	53 (17.7)

R, Reverse scored item; C, Condemnation; D, Death; E, Emptiness; F, Fate; G, Guilt; M, Meaninglessness.

**Table 4:** Existential anxiety prevalence.

Existential anxiety concern	*n* (%)
Death	288 (96)
Fate	259 (86.3)
Emptiness	237 (79)
Meaninglessness	278 (92.7)
Guilt	263 (87.7)
Condemnation	279 (93)

### Existential anxiety and its correlation with demographic characteristics

#### Age group

Participants aged 51-60 had the highest mean EA score (10.38 ± 1.245), indicating the most pronounced existential anxiety. This contrasts with the youngest group (younger than 20), which had the lowest mean EA score (7.60 ± 2.861). Notably, the 20-30 age group had a higher mean EA score (8.87 ± 2.457) compared to those under 20, but lower than the 31-40 (9.52 ± 2.042) and 41-50 (9.48 ± 2.294) groups.

#### Marital status

The study’s findings on existential anxiety (EA) scores about marital status reveal intriguing insights into how personal life circumstances might influence perceptions and anxieties regarding AI.

##### A- Divorced or widowed participants

Divorced or widowed participants exhibited the highest mean EA score (10.33 ± 0.816). This elevated level of anxiety could be linked to the life changes and uncertainties often associated with these statuses. Individuals who are divorced or widowed might face more concerns about financial security, social changes, and future uncertainties, potentially heightening their existential anxieties in the face of advancing technologies like AI.

##### B- Engaged individuals

In stark contrast, engaged individuals had the lowest mean EA score (7.86 ± 2.478). Engagement is typically a period of positive anticipation and planning for the future. This optimistic outlook might extend to their perceptions of AI, viewing it as less of a threat and more as a part of the evolving world they are preparing to navigate.

##### C- Married participants

Married participants had a higher mean EA score (9.62 ± 2.020) than single individuals (8.45 ± 2.691). The responsibilities and complexities associated with marital life, such as caring for family and managing joint finances, might contribute to a heightened sense of existential anxiety, especially in the context of potential disruptions caused by AI in the workplace or society.

##### D- Single individuals

**Figure 2:**
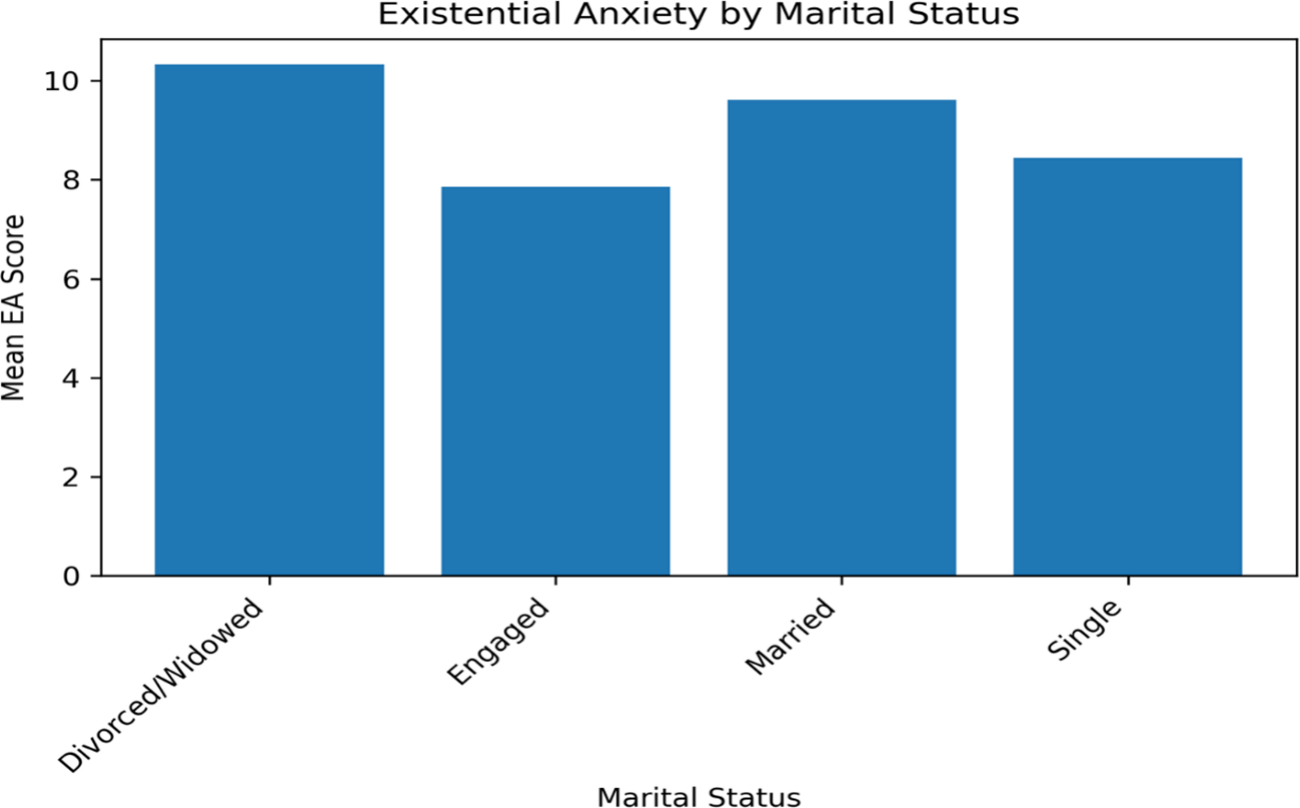
Existential anxiety and Marital status.

Single participants, having a lower mean EA score (8.45 ± 2.691) (**Table 5**; **Figure 2**), might experience less existential anxiety related to AI, due to fewer immediate family responsibilities or a more individual-centric lifestyle that allows for greater adaptability and flexibility in the face of technological changes.

**Table 5:** Existential Anxiety and Its Correlation with Demographic Characteristics.

Characteristic	*n*	EA Score Mean± (SD)	Death n (%)	Fate n (%)	Emptiness n (%)	Meaninglessness n (%)	Guilt n (%)	Condemnation n (%)
Age								
Younger than 20	73	7.60 ± 2.861	68 (93.2)	59 (80.8)	51 (69.9)	71 (97.3)	59 (80.8)	61
20-30	151	8.87 ± 2.457	146 (96.7)	124(82.1)	121 (80.1)	144 (95.4)	133(88.1)	143 (94.7)
31-40	23	9.52 ± 2.042	22 (95.7)	22 (95.7)	20 (87)	22 (95.7)	20 (87)	23 (100)
41-50	23	9.48 ± 2.294	23 (100)	23 (100)	18 (78.3)	21 (91.3)	22 (95.7)	22 (95.7)
51-60	24	10.38 ± 1.25	24 (100)	24 (100)	21 (87.5)	24 (100)	23 (95.8)	24 (100)
61 and above	6	8.50 ± 2.74	5 (83.3)	6 (100)	6 (100)	5 (83.3)	6 (100)	6 (100)
**P-value**			**0.001**	**0.002**	**0.023**	**0.079**	0.507	**0.001**
**Chi (*df)***			30.5 (10)	28.2 (10)	20.690 (10)	16.8(10)	9.3 (10)	28.77 (10)
Marital status								
Married	211	8.45 ± 2.691	202 (96)	172(81.5)	161 (76.4)	194 (92)	180(85.3)	192 (91)
Single	7	7.86 ± 2.478	6 (86)	5 (71.4)	7 (100)	6 (86)	6 (86)	6 (86)
Engaged	76	9.62 ± 2.020	74 (97.4)	75 (99)	63 (83)	72 (95)	71 (93.4)	75 (99)
Divorced or widowed	6	10.33 ± 0.82	6 (100)	6 (100)	6 (100)	6 (100)	6 (100)	6 (100)
**P-value**			**0.002**	**0.003**	0.118	0.914	0.432	**0.000**
**Chi (*df)***			20.96(6)	19.9 (6)	10.158 (6)	2.066 (6)	5.924 (6)	27.81 (6)
Employment status								
Employed/On payroll	175	8.42 ± 2.790	166 (95)	141 (81)	138 (79)	158 (90.3)	148 (85)	156 (89.1)
Unemployed/Not on payroll	97	9.09 ± 2.204	94 (97)	89 (92)	73 (75.3)	92 (95)	89 (92)	95 (98)
Retired	28	9.86 ± 1.693	28 (100)	28 (100)	26 (93)	28 (100)	27 (96.4)	28 (100)
**P-value**			0.130	**0.004**	0.294	0.247	0.291	**0.012**
**Chi (*df)***			7.118 (4)	15.5 (4)	4.937 (4)	5.4(4)	4.96(4)	12.825 (4)
Work Sector								
Government	168	8.40 ± 2.734	161 (96)	134 (80)	130 (77.4)	153 (91.1)	142 (85)	151 (90)
Private	93	9.39 ± 2.226	86 (92.5)	89 (96)	77 (83)	88 (95)	86 (92.5)	91 (98)
Student/Unemployed	39	8.87 ± 2.296	38 (97.4)	35(89.7)	30 (77)	37 (95)	35 (90)	37 (95)
**P-value**			**0.073**	**0.001**	0.435	0.736	0.359	**0.029**
**Chi (*df)***			8.550 (4)	18.5(4)	3.789 (4)	1.998 (4)	4.361 (4)	10.798 (4)
Years of Experience at Work/Study								
1-5 years	193	8.58 ± 2.625	186 (96)	159 (82)	147 (76)	178 (92)	169 (88)	177 (92)
6-10 years	32	8.37 ± 2.848	28 (88)	27 (84)	24 (75)	30 (94)	23 (72)	29 (91)
11-15 years	23	9.04 ± 2.205	23 (100)	21 (91)	21 (91)	21 (91)	22 (96)	22 (96)
16-20 years	7	9.29 ± 2.812	7 (100)	6 (86)	6 (86)	7 (100)	5 (71)	7 (100)
21 years or more	45	9.64 ± 2.047	44 (98)	45 (100)	39 (87)	42 (93)	44 (98)	44 (98)
**P-value**			**0.002**	**0.041**	**0.029**	0.412	**0.045**	**0.374**
**Chi (*df)***			24.889 (8)	16.072 (8)	17.138 (8)	8.221 (8)	15.844(8)	8.630 (8)
Occupation								
Health, science, and engineering	156	8.78 ± 2.448	149 (95.5)	128 (82.1)	125 (80.1)	148 (95)	136(87.2)	147 (94.2)
Education, Government, And Research	73	9.42 ± 2.273	69 (95)	73 (100)	58 (79.5)	68 (93.2)	67 (92)	70 (96)
Humanities	29	8.86 ± 2.682	29 (100)	27 (93.1)	25 (86.2)	28 (97)	27 (93.1)	26 (90)
IT and Technology	18	7.94 ± 2.960	18 (100)	13 (72.2)	12 (67)	15 (83.3)	15 (83.3)	15 (83.3)
Business and law	14	7.50 ± 2.504	13 (93)	10 (71.4)	10 (71.4)	12 (86)	11 (79)	13 (93)
Freelancers	10	6.90 ± 3.784	10 (100)	7 (70)	7 (70)	7 (70)	7 (70)	8 (80)
**P-value**			**0.011**	**0.001**	0.164	**0.010**	0.335	0.205
**Chi (*df)***			22.99 (10)	29.51 (10)	14.2 (10)	23.172 (10)	11.3 (10)	13.34 (10)

Bold text means significant P value.

#### Employment status and work sector

##### A- Employed/on payroll

Employed/payroll individuals had a mean EA score of 8.42 ± 2.790. This lower score suggests that having stable employment may mitigate existential anxiety regarding AI. This could be due to the security and regularity that come with employment, offering a sense of stability in the face of potential disruptions caused by AI.

##### B- Student/unemployed

Participants in the Student/Unemployed category showed a higher mean EA score (9.39 ± 2.226) than other sectors. This increased level of anxiety could be attributed to uncertainties regarding future career prospects and job security, especially in a rapidly changing technological landscape where AI is becoming more prevalent. Students and unemployed individuals might feel more vulnerable to the potential job market disruptions caused by AI advancements.

##### C- Government sector workers

In contrast, individuals in the Government sector had the lowest mean EA score (8.40 ± 2.734) among the three categories. The lower level of existential anxiety in this sector could be due to the perceived job security and stability often associated with government employment. Government jobs are often seen as less susceptible to rapid technological disruption compared to the private sector, alleviating some of the existential anxiety related to AI.

##### D- Private sector workers

The Private sector participants had a mean EA score of 8.87 ± 2.296, which lies between the Student/Unemployed and Government sectors. This intermediate level of anxiety could reflect a mix of stability and uncertainty in the private sector, where there is potential for both opportunities and threats from AI. Private sector employees might be experiencing a balance of excitement for innovation and fear of redundancy due to AI advancements.

### Years of experience at work/study

Participants with 6-10 years of experience exhibited the highest mean EA score (9.86 ± 1.693). This group, typically in the middle of their careers, might be experiencing heightened anxiety due to concerns about the rapid evolution of technology and its impact on job security and career progression. Their experience level places them in a position where they are established in their careers but may still feel vulnerable to technological disruptions. In contrast, those with 1-5 years of experience had a slightly lower mean EA score (9.09 ± 2.204), suggesting that individuals in the initial stages of their careers might be more open to or less aware of the potential threats posed by AI. This group might also be more adaptable to technological changes due to their recent entry into the workforce. Interestingly, the EA scores gradually decrease as the years of experience increase beyond 10 years. Participants with 11-15 years (9.04 ± 2.205), 16-20 years (9.29 ± 2.812), and 21 years or more (9.64 ± 2.047) showed lower levels of existential anxiety. This trend could be attributed to a higher level of career security and stability in these groups, as well as an adaptation to technological changes over time. Their extended experience might also provide them with a broader perspective on the evolution of technology and its impacts, reducing anxiety levels.

### Occupation

Existential anxiety (EA) scores across different occupations reveal significant differences, shedding light on how various professional fields perceive and respond to the potential impacts of AI.

#### A- Education, government, and research sector

Individuals in the Education, Government, and Research Sector exhibited the highest mean EA score among the surveyed occupations (9.42 ± 2.273). This heightened anxiety level could be attributed to concerns about how AI and technology might revolutionize the educational landscape. Educators might worry about the replacement of traditional teaching methods with AI-driven tools or the challenge of integrating AI into curricula while maintaining educational quality and personal interaction.

#### B- Health sciences and engineering

Participants in Health sciences and engineering had a lower mean EA score (8.78 ± 2.448) compared to those in Education. This could reflect a more nuanced understanding or familiarity with AI and its applications within these fields. Professionals in health science and engineering are often at the forefront of applying innovative technologies, which might reduce anxiety due to a better grasp of AI’s limitations and potential.

#### C- Freelancers, business and law, and humanities

**Figure 3:**
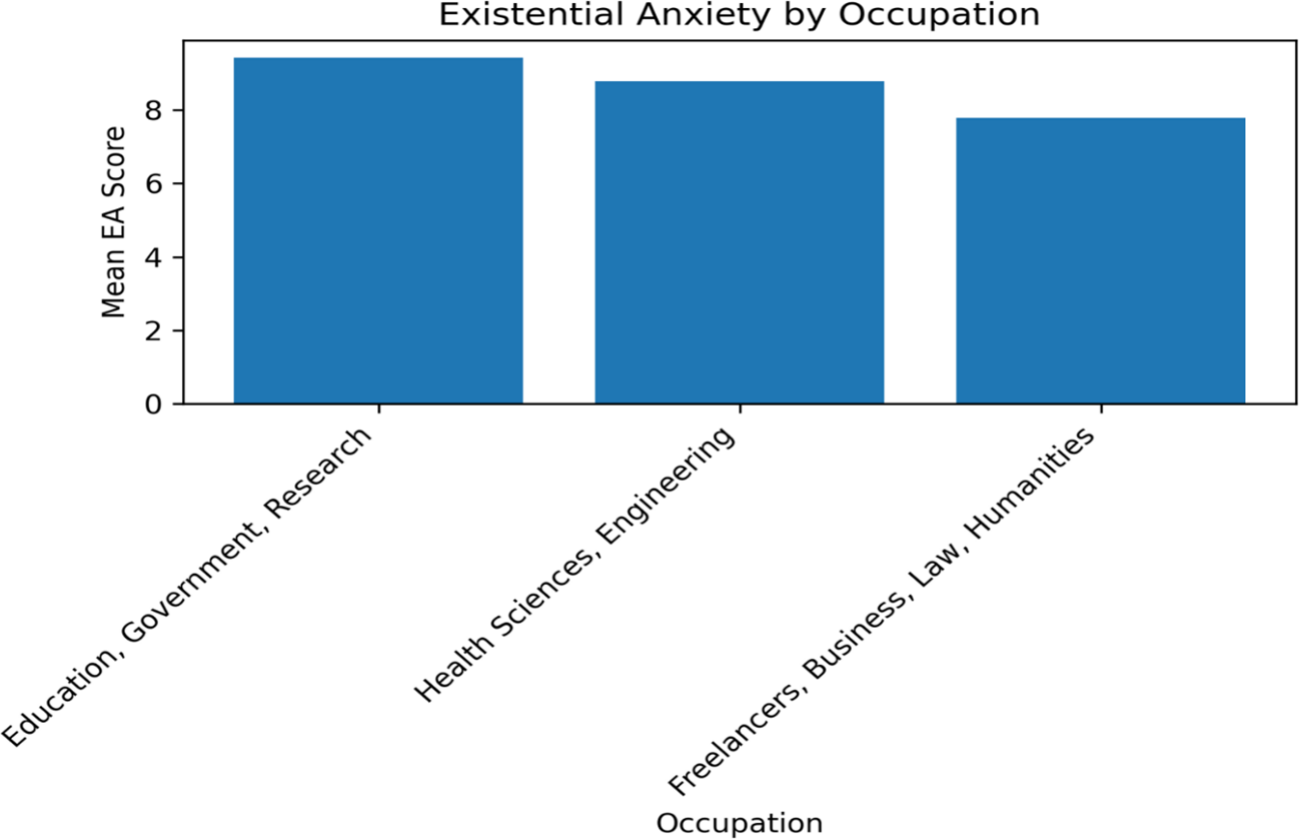
Existential anxiety and Occupation.

The lowest mean EA scores were observed in Freelancers (6.90 ± 3.784), Business and Law (7.50 ± 2.504), and Humanities (7.94 ± 2.960) (**Table 5**; **Figure 3**). These lower scores suggest a lesser degree of existential anxiety regarding AI in these occupations. Freelancers might perceive AI as a tool that can open new opportunities or enhance productivity, rather than a threat to job security. Professionals in Business and Law might view AI as a beneficial asset that can aid in data analysis, research, and administrative tasks, thereby enhancing their work rather than replacing it. Individuals in the Humanities might feel less threatened by AI, given the nature of their work which often requires critical thinking, creativity, and emotional intelligence – skills that are currently less replicable by AI.

### Existential anxiety and its correlation with artificial intelligence use

#### Awareness of AI development

Participants who were aware of AI and had tried Chat-GPT had a mean EA score of 8.65 ± 2.724 (65 participants). Those who were aware but had not tried Chat-GPT had a slightly higher mean EA score of 9.09 ± 2.638 (92 participants). Participants who were not aware of AI developments like Chat-GPT had a mean EA score similar to the first group, at 8.62 ± 2.435 (143 participants). The data suggests a subtle correlation between the level of awareness and interaction with AI and existential anxiety. Those who were aware of AI but had not interacted with it expressed slightly higher anxiety levels.

### Frequency of AI use in career

Participants who rarely used AI (1-3 times/month) had a mean EA score of 8.26 ± 2.870 (50 participants). Those who used AI often (once weekly) had a mean EA score of 8.81 ± 3.291 (16 participants). Participants using AI constantly (every other day) had a lower mean EA score of 7.60 ± 2.302 (5 participants). Participants who used AI always (daily) reported the lowest mean EA score of 6.50 ± 2.121 (2 participants). A notable trend is observed in the frequency of AI use in one’s career and EA scores. More frequent users of AI, particularly those using it daily, showed lower existential anxiety scores. This could indicate that familiarity and regular interaction with AI might reduce anxiety related to its impacts.

### Fear of AI replacing jobs

Participants who strongly agreed with the fear that AI may soon replace their job had a high mean EA score of 9.35 ± 2.315 (65 participants). Those who partially agreed with this fear had a marginally higher mean EA score of 9.39 ± 2.045 (70 participants). Participants who were neutral towards this possibility had a lower mean EA score of 8.97 ± 2.568 (65 participants). Individuals who partially disagreed with the fear of AI replacing their jobs had a mean EA score of 8.44 ± 2.534 (71 participants). Those who strongly disagreed showed the lowest mean EA score of 5.85 ± 2.588 (26 participants) (**Table 6**; **Figure 1**). Concerning the fear of AI replacing jobs, higher existential anxiety scores were observed in participants who strongly or partially agreed with this fear. This indicates that concerns about job security in the face of advancing AI technology may contribute significantly to existential anxiety. Conversely, those who disagreed with the fear of AI replacing their jobs exhibited significantly lower existential anxiety scores, suggesting a more optimistic or unconcerned outlook towards AI’s impact on employment.

**Table 6:** Existential anxiety and its correlation with artificial intelligence use.

Questions	*n*	Mean± (SD) EA Score	Death n (%)	Fate n (%)	Emptiness n (%)	Meaninglessness n (%)	Guilt n (%)	Condemnation n (%)
1- Are you aware of the recent development of Artificial Intelligence (AI), for example, Chat-GPT?								
Yes, and I have tried the chatbot	65	8.65 ± 2.724	60 (92)	58 (89)	52 (80)	60 (92)	52 (80)	61 (94)
Yes, but I haven’t tried it yet	92	9.09 ± 2.638	90 (98)	76 (83)	73 (79)	84 (91)	83 (90)	86 (93)
No	143	8.62 ± 2.435	138 (97)	124 (87)	112 (78)	134 (94)	128 (90)	132 (92)
**P-value**			**0.037**	**0.041**	0.225	**0.371**	0.165	0.726
**Chi (*df)***			10.230 (4)	9.992(4)	5.667 (4)	4.265 (4)	6.490 (4)	2.055 (4)
2- If you answered yes to the previous question, how often do you use AI in your career?								
Rarely (1-3times\month)	50	8.26 ± 2.870	44 (88)	39 (78)	41 (82)	44 (88)	40 (80)	37 (74)
Often (once weekly)	16	8.81 ± 3.291	16 (100)	15 (94)	12 (75)	13 (81.3)	14 (88)	13 (81.3)
Constantly (every other day)	5	7.60 ± 2.302	4 (80)	5 (100)	4 (80)	4 (80)	4 (80)	4 (80)
Always (daily)	2	6.50 ± 2.121	2 (100)	1 (50)	1 (50)	2 (100)	2 (100)	2 (100)
**P-value**			0.124	0.218	0.699	0.083	0.656	0.813
**Chi (*df)***			12.659 (8)	10.714 (8)	5.533 (8)	13.936 (8)	5.924 (8)	4.462 (8)
3- I have a genuine fear that in the near future Artificial Intelligence may replace me at my job								
Strongly Agree	65	9.35 ± 2.315	63 (97)	56 (86.2)	57 (88)	63 (97)	61 (94)	61 (94)
Partially Agree	70	9.39 ± 2.045	68 (97.1)	63 (90)	53 (76)	68 (97.1)	64 (91.4)	69 (99)
Neutral	65	8.97 ± 2.568	64 (99)	59 (91)	56 (86.2)	60 (92.3)	59 (91)	62 (95.4)
Partially Disagree	71	8.44±​​2.534	67 (94.4)	59 (83.1)	55 (77.5)	66 (93)	55 (77.5)	64 (90.1)
Strongly Disagree	26	5.85 ± 2.588	23 (88.5)	18 (69.2)	13 (50)	18 (69.2)	21 (81)	20 (77)
**P-value**			0.264	0.095	**0.000**	**0.000**	**0.000**	**0.000**
**Chi (*df)***			10.015 (8)	13.538 (8)	32.049 (8)	39.108 (8)	34.04 (8)	33.04 (8)

Bold text means significant P value.

## Discussion

This study’s exploration into the existential anxiety (EA) associated with AI development has highlighted a complex array of concerns among participants. Particularly striking is the high prevalence of existential concerns, with the fear of meaninglessness reported by 92.7% of participants. This anxiety, as noted by Yalom (12), is not just about personal relevance in the face of AI advancements, but also reflects deeper philosophical and societal questions.

The pervasive sense of meaninglessness can be contextualized within a philosophical perspective that emphasizes a change in basic assumptions in societal values. According to the World Economic Forum (WEF), the AI evolution will disrupt 85 million jobs globally between 2020 and 2025 and create 97 million new job roles, thereby necessitating about 40 per cent of the global workforce to reskill in the next three years (13). In a world that increasingly defines humans by their economic value and now threatens to replace them with AI, we face a potential catastrophe. The new challenge upon humanity is not merely an ability to establish technological advancements but to redefine and reaffirm human values. This perspective aligns with Frankl’s (14) assertion on the importance of finding meaning and purpose beyond economic or occupational status. The relevance of this perspective in the context of AI is further explored by Solomon et al. (15), who discuss how technological changes challenge traditional sources of meaning. Our study findings suggest that human worth should not be determined by economic productivity or financial status but by moral superiority and ethical considerations.

Guilt and condemnation, experienced by 87.7% and 93% of participants, tie into the moral and ethical dimensions of existential anxiety. Greenberg et al. (16) offer insights into how technological advancements, such as AI, can trigger deep-seated ethical dilemmas and guilt, contributing to existential concerns. Furthermore, Kierkegaard’s (17) exploration of existential despair offers a philosophical foundation for understanding these anxieties in the face of AI’s potential societal upheaval. May’s (18) work on the importance of myth in providing a sense of meaning and structure in a rapidly changing world further complements this understanding.

The situation we are currently facing can be likened to a new, revolutionized industrial revolution, similar to Heidegger’s (19) concept of ‘thrownness’ into new existential conditions. Just as the original Industrial Revolution saw factory workers replaced by machines, the AI revolution threatens to eclipse the role of humans in an unprecedented manner. History, as discussed by Harari (20), is replete with examples of technological advancement supplanting human roles, but never before has humanity faced a challenge where a single technological force has the potential to replace every aspect of human endeavour.

This study’s findings are a clarion call, indicating the onset of a potential psychological pandemic that demands immediate and concerted efforts to address. If left unchecked, the existential anxiety stemming from these technological advancements could lead to societal upheaval. As Bostrom (21) warns, addressing these concerns is crucial, not just for the current generation, but to safeguard the psychological well-being and value system of future societies. This manuscript emphatically recommends that individuals in positions of influence, such as policymakers, academicians, educators, and notable public figures, focus their attention on the significant peril presented by the threat of this psychiatric pandemic. This phenomenon possesses the capacity to remain covert, eluding public awareness; however, the potential repercussions that are of concern could profoundly affect our societies. The conviction held by the authors is that the current study has merely laid the groundwork for a research domain that necessitates comprehensive exploration among all groups including those with diagnosed mental health conditions and correlating the findings to further elaborate the effect in an inclusive manner to all. This entails examining methodologies to halt the spread, devising therapeutic strategies, and augmenting institutional support in educational and psychological spheres.

### Study strengths and limitations

The study addresses a timely and significant topic, exploring the intersection of technology and human psychology. Utilization of a structured survey to systematically gather data on existential anxieties related to AI. This novel and interesting study finding encompassed a diverse population and findings are important as the result addressed the existential anxieties in the age of AI. This study enables holistic findings of the interplay between AI-based technological advancements and human experience, nurturing debates, and actions to direct these existential concerns responsibly. The study findings can drive an innovative approach to creating AI systems that align with human values and foster adaptation strategies to coexist harmoniously with advanced technology. To our knowledge, this is the first study to investigate the psychological impact of the rapid development of AI. The limitations of this study include a small sample size of 300 participants and the geographical and cultural specificity that may stand in the way of generalizing the results. The questionnaire was self-administered so we cannot rule out response bias. Another limitation is that individuals who are divorced or widowed might face more concerns about financial security, social changes, and future uncertainties. The people in government jobs are often seen as less susceptible to rapid technological disruption compared to the private sector. These factors may alleviate existential anxiety related to AI.

## Conclusions

The public has concerns including unpredictability, a sense of emptiness, anxiety, guilt over potential AI-related catastrophes, fear of condemnation due to ethical dilemmas in AI, and apprehensions about humanity’s future in an AI-dominated era. The findings addressing existential anxieties in the age of AI require a multidisciplinary approach. This approach should integrate technological innovation with psychological, philosophical, and ethical considerations, emphasizing the importance of human values in a rapidly evolving technological landscape.

## Data availability statement

The raw data supporting the conclusions of this article will be made available on reasonable request to corresponding author.

## Ethics statement

Institutional Review Board, College of Medicine Research Centre, King Saud University, Riyadh, Saudi Arabia, approved the protocol (Ref. No. 23/0445/IRB). The studies were conducted in accordance with the local legislation and institutional requirements. The participants provided their written informed consent to participate in this study.

## Author contributions

JA: Data curation, Validation, Writing – original draft, Writing – review & editing. AMB: Writing – original draft, Data curation, Formal analysis. NS: Data curation, Formal analysis, Investigation, Writing – original draft. WS: Investigation, Methodology, Writing – original draft. SM: Writing – original draft, Conceptualization, Supervision, Writing – review & editing.

## References

[1] Tomaszek K Muchacka-Cymerman A. Thinking about My Existence during COVID-19, I Feel Anxiety and Awe-The Mediating Role of Existential Anxiety and Life Satisfaction on the Relationship between PTSD Symptoms and Post-Traumatic Growth. Int J Environ Res Public Health. (2020) 17:7062. doi: 10.3390/ijerph17197062 32992515 PMC7579162

[2] Tomaszek K Muchacka-Cymerman A. Student burnout and PTSD symptoms: the role of existential anxiety and academic fears on students during the COVID-19 pandemic. Depress Res Treat. (2022), 6979310. doi: 10.1155/2022/6979310 35096425 PMC8796705

[3] Abulof U Le Penne S Pu B. The pandemic politics of existential anxiety: Between steadfast resistance and flexible resilience. Int Polit Sci Rev. (2021) 42:19251212110020. doi: 10.1177/01925121211002098

[4] Scrima F Miceli S Caci B Cardaci M. The relationship between fear of COVID-19 and intention to get vaccinated. The serial mediation roles of existential anxiety and conspiracy beliefs. Pers Individ Dif. (2021), 111188. doi: 10.1016/j.paid.2021.111188 34393312 PMC8354796

[5] Brauner P Hick A Philipsen R Ziefle M. What does the public think about artificial intelligence? A criticality map to understand bias in the public perception of AI. Front Comput Sci. (2023) 5:1113903. doi: 10.3389/fcomp.2023.1113903

[6] Yigitcanlar T Degirmenci K Inkinen T. Drivers behind the public perception of artificial intelligence: insights from major Australian cities. AI Soc. (2022) . 3:1–21. doi: 10.1007/s00146-022-01566-0 PMC9527736 36212229

[7] Nussberger AM Luo L Celis LE Crockett MJ. Public attitudes value interpretability but prioritize accuracy in Artificial Intelligence. Nat Commun. (2022) 13:5821. doi: 10.1038/s41467-022-33417-3 36192416 PMC9528860

[8] Tillich P. The courage to be. Yale University Press (2008). Available at: https://philarchive.org/archive/STETCT-13.

[9] Weems CF Costa NM Dehon C Berman SL. Paul Tillich's theory of existential anxiety: A preliminary conceptual and empirical examination. Anxiety Stress Coping. (2004) 17:383–99. doi: 10.1080/10615800412331318616

[10] Berman SL Weems CF Stickle TR. Existential anxiety in adolescents: Prevalence, structure, association with psychological symptoms and identity development. J Youth adolescence. (2006) 35:285–92. doi: 10.1007/s10964-006-9032-y

[11] To SM Chan WC. Psychometric evaluation of the Chinese version of the Existential Anxiety Questionnaire in a sample of Chinese adolescents living in Hong Kong. In Child Youth Care Forum. (2016) 45:487–503. doi: 10.1007/s10566-016-9347-0

[12] Yalom ID. Existential Psychotherapy. New York, USA: Basic Books (1980). Available at: www.hachettebookgroup.com/psychotherapy/9780465021475/?lens=basic-books.

[13] World Economic Forum. The Future of Jobs Report 2023. Geneva: World Economic Forum (2023). Available at: https://www.weforum.org/publications/the-future-of-jobs-report-2023/.

[14] Frankl VE. Man's Search for Meaning. Boston, MA: Beacon Press (1946/2006). Available at: https://www.beacon.org/Mans-Search-for-Meaning-P602.aspx.

[15] Solomon S Greenberg J Pyszczynski T. The Worm at the Core: On the Role of Death in Life. Random House (2004). Available online at: https://www.amazon.com/Worm-Core-Role-Death-Life/dp/1400067472.

[16] Greenberg J Solomon S Pyszczynski T. The causes and consequences of a need for self-esteem: A terror management theory. In: Baumeister RF, editor. Public self and private self. New York, NY: Springer-Verlag (1986). p. 189–212.

[17] Kierkegaard S. The Concept of Anxiety: A Simple Psychologically Orienting Deliberation on the Dogmatic Issue of Hereditary Sin. Princeton University Press (1844/1980).

[18] May R. The Cry for Myth. W. W. Norton & Company (1991). Available at: https://www.amazon.com/Cry-Myth-Rollo-May/dp/0393331776.

[19] Heidegger M. Being and Time. Harper & Row (1927/1962). Available at: http://pdf-objects.com/files/Heidegger-Martin-Being-and-Time-trans.-Macquarrie-Robinson-Blackwell-1962.pdf.

[20] Harari YN. Homo Deus: A Brief History of Tomorrow. Harper (2017). Available at: https://www.amazon.com/Homo-Deus-Brief-History-Tomorrow/dp/0062464310.

[21] Bostrom N. Superintelligence: Paths, Dangers, Strategies. Oxford University Press (2014) p. 32–5.

